# Associations of deep medullary veins with vascular risk factors, laboratory indicators, and cerebral small vessel disease: A population‐based study

**DOI:** 10.1002/brb3.2974

**Published:** 2023-04-09

**Authors:** Yu Tian, Shan Li, Yingying Yang, Xueli Cai, Jing Jing, Suying Wang, Xia Meng, Lerong Mei, Aoming Jin, Dongxiao Yao, Tiemin Wei, Yongjun Wang, Yuesong Pan, Yilong Wang

**Affiliations:** ^1^ Department of Neurology Beijing Tiantan Hospital Capital Medical University Beijing China; ^2^ China National Clinical Research Center for Neurological Diseases Beijing China; ^3^ Cerebrovascular Research Lab The Fifth Affiliated Hospital of Wenzhou Medical University Lishui China; ^4^ Department of Neurology The Fifth Affiliated Hospital of Wenzhou Medical University Lishui China; ^5^ Department of Cardiology The Fifth Affiliated Hospital of Wenzhou Medical University Lishui China

**Keywords:** aging, cerebral small vessel disease, deep medullary veins, enlarged perivascular spaces, white matter hyperintensity

## Abstract

**Objective:**

Deep medullary veins (DMVs) were not considered a typical marker of cerebral small vessel disease (CSVD) due to limited understanding of their involvement in pathology of CSVD. This study aimsto investigate potential vascular risk factors for DMVs and their associations with CSVD.

**Methods:**

In total, 1909 community‐dwelling participants were included in this analysis. Demographic, clinical, laboratory, and imaging data were collected. DMV scores (0–18) werecalculated as the sum of bilateral frontal, parietal, and occipital regional scores using a semiquantitative visual scale (0–3). The presence, total burden, and imaging markers of CSVD were assessed. Linear regression analyses were conducted to explore potential vascular factors for DMV scores. Binary and ordinal logistic regression analyses were performed to investigate the associations of DMV scores with CSVD and its markers.

**Results:**

Mean age was 61.8 (SD 6.5) years, and 1027 (53.8%) of participants were men. The median DMV scores were14 (IQR 12–16). DMV scores wererelated to age, male sex, body mass index, diastolic blood pressure, hypercholesterolaemia, atrial fibrillation, current drinking, total cholesterol, triglycerides, low‐density lipoprotein, hemoglobin A1c, leukocytes, lymphocytes, hemoglobin, and platelets (*p* < .05). DMV scores wereassociated with the presence and total burden of CSVD (Rothwell's scale), modified white matter hyperintensity burden, and enlarged perivascular spaces in centrum semiovale (*p* < .05). However, these associations between DMV scores and CSVD disappeared after adjusting for potential confounders.

**Conclusion:**

Several conventional vascular factors were associated with DMVs. The relationship between DMVs and CSVD was vulnerable, suggesting decreased visible and discontinuous DMVs may differ mechanistically from traditional markers of CSVD.

## Graphical abstract

Among 1909 community‐dwelling participants, DMV scores wererelated to age, male sex, body mass index, diastolic blood pressure, hypercholesterolaemia, atrial fibrillation, current drinking, total cholesterol, triglycerides, low‐density lipoprotein, hemoglobin A1c, leukocytes, lymphocytes, hemoglobin, and platelets (*p* < .05). DMV scores wereassociated with the presence and total burden of CSVD (Rothwell's scale), modified white matter hyperintensity burden, and enlarged perivascular spaces in centrum semiovale (*p* < .05). However, these associations between DMV scores and CSVD disappeared after adjusting for potential confounders.

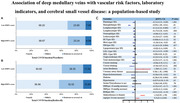

## 1 INTRODUCTION

Cerebral small vessel disease (CSVD) is caused by microvascular structural and functional dysfunction, and accounts for a quarter of stroke and almost half of vascular cognitive impairment and dementia (Ter Telgte et al., 2018). The mechanisms of CSVD involving small arteries, arterioles, and capillaries, such as arteriolosclerosis, neurovascular unit dysfunction, blood–brain barrier damage, and hemodynamic instability, were investigated thoroughly. Venous collagenosis (VC), characterized by collagen deposition within venous walls, narrowed lumen, and occlusion of venules (Moody et al., 1995), was longoverlooked. This may partly be due to difficulties of visualizing VC that is only clearly identified by pathology. Furthermore, thickened venous walls may easily be mistaken for arteriolar hyalinization. Until recently, the underlying role of venules has been gradually established in CSVD pathogenesis (Pantoni, 2010).

With the advent of iron‐sensitive susceptibility‐weighted imaging (SWI), deep medullary veins (DMVs) can now be assessed in vivo as characteristic low‐signal‐intensity lines perpendicular to the lateral ventricles. As only visible small blood vessels on conventional 1.5 or 3.0 Tesla magnetic resonance imaging (MRI), interrupted, tortuous, and reduced DMVs drawconsiderable interest. Currently, there is still debate as to whether visible DMVs on SWI are novel radiological markers for CSVD. A community‐based study reported that several common vascular risk factors, such as hypertension and diabetes, did not seem to have robust associations with DMVs (Ao et al., 2021). Moreover, the relationshipsof DMVs withtotal burden and traditional imaging features of CSVD were based on studies with small sample sizes (Xu et al., 2020). To date, there is insufficient evidence to support the vital role of DMVs in the progress of general aging and their far‐reaching effects on CSVD.

Using cross‐sectional data from a baseline survey of a population‐based cohort, this study aimsto provide a comprehensive insight into the potential role of DMVs during the aging process. First, we investigated the associations between visible DMVs on SWI and common vascular risk factors. Moreover, we explored the relationships between DMVs and the presence, severity, and conventional imaging markers of CSVD.

## 2 MATERIALS AND METHODS

### 2.1 Study design and participants

All data in this study were obtained from the Polyvascular Evaluation for Cognitive Impairment and Vascular Events (PRECISE) study. The details of the PRECISE cohorthave been published elsewhere (Pan et al., 2021). Briefly, the PRECISE study is an ongoing population‐based prospective cohort study of community‐dwelling residents that aimsto comprehensively evaluate multivascular events and cognitive function. A total of 3067 residentsaged 50−75 years were enrolled at the baseline interviews in the PRECISE study based on cluster sampling from six villages and four communities of Lishui City, Zhejiang Province, China. In the present cross‐sectional analysis, we analyzed the baseline data for all participants with qualified MRI data.

### 2.2 Standard protocol approvals, registrations, and participants’ consent

The PRECISE study protocol was approved by the Ethics Committee of Beijing Tiantan Hospital (IRB approval number: KY2017‐010‐01) and Ethics Committee of the Fifth Affiliated Hospital of Wenzhou Medical University(IRB approval number: 2016–42) (Pan et al., 2021). All participants or their representatives provided written informed consent before enrolment in the trial.

### 2.3 Clinical data collection

Centralized trained research coordinators collected clinical characteristics and examinations at baseline via face‐to‐face interviewsaccording to a standard data collection protocol, including age, sex, body mass index (BMI), current smoking and drinking statuses, systolic and, diastolic blood pressure (SBP and DBP), medical history, medication use, and the Montreal Cognitive Assessment (MoCA). Fasting blood samples were collected to assess several biomarkers, including routine blood examination (such as leukocytes, neutrophils, lymphocytes, monocytes, hemoglobins, and platelets), blood lipid profiles (including total cholesterol [TC], triglycerides [TG], high‐density lipoprotein [HDL], low‐density lipoprotein [LDL], and lipoprotein [a]), homocysteine, fasting blood glucose (FBG), and hemoglobin A1c (HbA1C).

### 2.4 MRI acquisition

All participants underwent brain structural MRI, including T1‐weighted (T1w), T2‐weighted (T2w), fluid‐attenuated inversion recovery (FLAIR), diffusion‐weighted imaging (DWI), and SWI in the same 3.0 Tesla MRI scanner (Ingenia 3.0T; Philips, Best, The Netherlands). The detailed imaging parameters of MRI sequences are presented in Table S1. All imaging data were stored in Digital Imaging and Communications in Medicine (DICOM) format on disks and centrally analyzed at Beijing Tiantan Hospital.

### 2.5 DMV interpretation and evaluation

The degree of DMVs was assessed using a previously published semiquantitative visual scale (Zhang et al., 2017). DMVs were evaluated as decreased signal intensity lines around the lateral ventricles on SWI. The location of DMVs was defined as the distance from the level of the lateral ventricles above the basal ganglia (BG) to the level at which the lateral ventricles disappeared, according to a previous anatomical report (Lee et al., 1996). The regions of interest of the DMVs were separated into frontal, parietal, and occipital regions, which were estimated bilaterally. A semiquantitative visual inspection of DMVs was applied to evaluate each region: (1) 0 points: each vein was continuous and exhibited a homogeneous signal; (2) 1 point: each vein was continuous, but at least one vein exhibited an inhomogeneous signal; (3) 2 points: at least one vein exhibited an inhomogeneous signal and with spot‐like decreased intensity; and (4) 3 points: no vein presented continuously (Zhang et al., 2017). The total score of visible DMVs on SWI was the sum of the six regional scores (range: 0–18).

### 2.6 CSVD interpretation and evaluation

Traditional CSVD markers were definedaccording to the Standards for Reporting Vascular Changes on Neuroimaging Criteria (STRIVE) (Wardlaw et al., 2013). Each marker of CSVD was assessed by two bridle‐wise neurologists (M. Zhou, Y. Chen, J. Pi, M. Zhao, and Y. Tian, with each neurologist being responsible for two features) blinded to the participants’ clinical information. When inconsistent results were obtained, another senior neurologist (Y. Yang) blinded to the initial results decided the final assessment. White matter hyperintensity (WMH) was defined as an increased signal on FLAIR and was classified as periventricular WMH (P‐WMH) and deep WMH (D‐WMH). The severity of WMH was determined according to the Fazekas scale (Fazekas et al., 1987). Lacunae were defined as rounded or ovoid subcortical lesions (3−15 mm) with a cerebrospinal fluid signal on T1w and FLAIR, usually with a hyperintense rim. Enlarged perivascular spaces (EPVSs) were defined as several punctate or linear lesions located in the BG or centrum semiovale (CSO), with intensities similar to those of cerebrospinal fluid on T2w and FLAIR, and were rated on a previously validated semiquantitative scale (none, 0; mild, 1–10; moderate, 11–20; frequent, 21–40; severe, >40) (Potter et al., 2015). Cerebral microbleeds (CMBs) were manually quantified by identifying ≤10 mm small round or ovoidof decreased signal on SWI. CMBs burden was classified as grade 0 (absent CMB), grade 1 (1–4 CMBs), and grade 2 (≥5 CMBs). The Global Cortical Atrophy (GCA) scale was used to assess brain atrophy.

In this study, the presence and severity of CSVD were assessed using two previously published CSVD scales by Wardlaw et al. and Rothwell et al., respectively. On Wardlaw's scale (0–4), 1 point was awarded for each of the following: (1) WMH burden, defined as confluent D‐WMH (Fazekas scores 2 or 3) or irregular P‐WMH extending into the deep white matter (Fazekas scores3), (2) one or more lacunae, (3) one or more CMBs, and (4) moderate‐to‐severe BG‐EPVS (Staals et al., 2014). On Rothwell's scale (0–6), 1 point was awarded for each of the following: (1) moderate modified WMH burden (total Fazekas scores of P‐WMH and D‐WMH 3 or 4), (2) one or more lacunae, (3) one tofour CMBs, and (4) moderate‐to‐severe BG‐EPVS; 2 points were awarded for each of the following: (1) five or more CMBs and (2) severe modified WMH burden (total Fazekas scores of P‐WMH and D‐WMH 5 or 6) (Lau et al., 2017). Total CSVD burden of 0 points was defined as absence of CSVD; otherwise, presence was defined.

### 2.7 Statistical analysis

Categorical variables are presented as percentages, and continuous variables are presented as means with standard deviations (SDs) or medians with interquartile ranges (IQRs). The participants were divided into two groups according to the median DMV scores. Clinical characteristics were compared between the included participants and those excluded from this study and between the two groups divided by the median of DMVs using Chi‐square test for categorical variables and *t*‐test or Wilcoxon rank‐sum test for continuous variables, as appropriate.

We used linear regression analyses to examine the associations of DMVs with potential vascular risk factors, in which the DMV scores weretreated as the dependent variable and vascular risk factors were treated as independent variables. The beta coefficients and 95% confidence intervals (CIs) were calculated based on each DMV scores increment. We further investigated statistically significant differences in the interaction between age and sex.

Ordinal logistic regression analyses were used to investigate the associations of DMVs with total CSVD burden, modified WMH burden, and CMBs burden. Common odds ratios (cORs) and 95% CIs were calculated based on each DMV scores increment. Binary logistic regression analyses were used to explore the associations between DMVs and the presence of CSVD, WMH burden, BG‐EPVS, CSO‐EPVS, lacunae, CMBs, and brain atrophy. ORsand 95% CIs were calculated based on each DMV scores increment. In the logistic regression analyses, the traditional neuroimaging features of CSVD were treated as dependent variables, and DMV scores weretreated as the independent variable. To eliminate the underlying confounding bias in a cross‐sectional observational study, we adjusted for potential covariates in the three models. In model 1, we did not adjust for any covariates; in model 2, we adjusted for age and sex; and in model 3, we adjusted for age, sex, BMI, stroke or transient ischemic attack, hypertension, diabetes mellitus, hypercholesterolaemia, coronary artery disease, atrial fibrillation, current drinking andsmoking, and MoCA scores.

All statistical analyses were performed using SAS software (version 9.4; SAS Institute, Inc., Cary, NC, USA). A two‐sided *p*‐value of <.05 was defined as the threshold for statistical significance.

## 3 RESULTS

### 3.1 Clinical and neuroimaging characteristics

**FIGURE 1:**
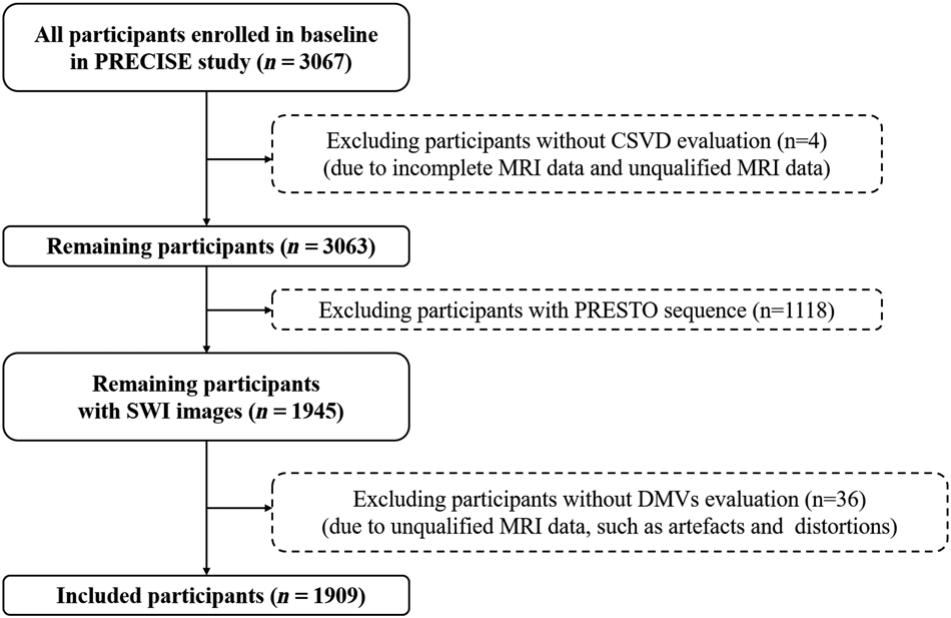
Flowchart. PRECISE, the Polyvascular Evaluation for Cognitive Impairment and Vascular Events (PRECISE) study; CSVD, cerebral small vessel disease; PRESTO, PRinciples of EchoShifting with a Train of Observation; DMVs, deep medullary veins; MRI, magnetic resonance imaging.

A total of 1909 community‐dwelling adults with available MRI data were enrolled in the current study (Figure 1). Mean age was 61.8 (SD 6.5) years, and 1027 (53.8%) of participants were men. Compared to excluded participants, participants who were included were older and had slightly higher levels of BMI and SBP, hada higher proportion of hypertension, diabetes, hypercholesterolaemia, current drinking, current smoking, and lipid‐lowering medication, antidiabetic medicationand had more CMBs, moderate‐to‐severe CSO‐EPVS, and brain atrophy (Tables S2 and S3).

Among the 1909 included individuals, the median DMV scores were14 (IQR 12–16). The demographics and clinical characteristics of the participants with lower and higher DMV scores are provided in Table 1. Age, sex, BMI, DBP, diabetes, current smoking, current drinking, lipid profile level, FBG, homocysteine, HBA1c, routine blood examination, lipid‐lowering medication, and MoCA scores differed between the two groups.

**TABLE 1:** Baseline characteristics of all included participants according the DMV scores

Characteristic	Low DMV scores group (*n* = 1044)	High DMV scores group (*n* = 865)	*p*‐value
Sociodemographics			
Age (years), mean ± SD	61.4 ± 6.3	62.2 ± 6.7	.008
Sex (male), *n* (%)	468 (44.8)	559 (64.6)	<.001
BMI (kg/m^2^), median (IQR)	24.2 ± 2.8	23.6 ± 3.1	<.001
SBP (mmHg), mean ± SD	129.5 (119.5–140.0)	128.5 (117.5–140.0)	.32
DBP (mmHg), mean ± SD	76.0 (70.5–82.0)	73.5 (67.5–79.5)	<.001
Vascular risk factors, *n* (%)			
Stroke/TIA	29 (2.8)	34 (3.9)	.16
Hypertension	480 (46.0)	375 (43.4)	.25
Diabetes mellitus	274 (26.3)	174 (20.1)	.002
Hypercholesterolemia	246 (23.6)	181 (20.9)	.17
Coronary artery disease	4 (0.4)	5 (0.6)	.78
Atrial fibrillation	8 (0.8)	11 (1.3)	.27
Current drinking	224 (21.5)	105 (12.1)	<.001
Current smoking	240 (23.0)	117 (13.5)	<.001
Laboratory data, median (IQR)			
Total cholesterol (mmol/L)	5.4±1.0	5.3±1.0	.004
Triglycerides (mmol/L)	1.6 (1.1–2.3)	1.4 (1.0–2.0)	<.001
HDL‐C (mmol/L)	1.3 (1.1–1.5)	1.35 (1.2–1.6)	<.001
LDL‐C (mmol/L)	2.7 (2.3–3.3)	2.6 (2.2–3.2)	.01
Lipoprotein (a) (mg/L)	66.0 (36.0–160.0)	69.0 (37.0–150.0)	.49
FBG (mmol/L)	5.6 (5.3–6.2)	5.5 (5.1–6.0)	<.001
HbA1c (%)	5.8 (5.5–6.2)	5.7 (5.5–6.0)	<.001
HCY (mmol/L)	11.2 (9.3–13.9)	10.7 (9.0–12.9)	<.001
Leukocyte (10^9^/L)	6.1 (5.2–7.2)	5.7 (5.0–6.8)	<.001
Neutrophil (10^9^/L)	3.3 (2.7–4.2)	3.10 (2.60–3.90)	<.001
Lymphocyte (10^9^/L)	2.0 (1.70–2.4)	1.90 (1.6–2.3)	<.001
Monocyte (10^9^/L)	0.4 (0.4–0.5)	0.4 (0.3–0.5)	<.001
Hemoglobin (g/L)	146.0 (138.0–154.0)	137.0 (130.0–144.0)	<.001
Platelet (10^9^/L)	209.0 (176.0–241.0)	202.0 (168.0–238.0)	.006
Medication use, *n* (%)			
Antihypertensive	288 (27.6)	239 (27.6)	.98
Lipid‐lowering	41 (3.9)	52 (6.0)	.04
Antiplatelet	28 (2.7)	31 (3.6)	.26
Anticoagulant	2 (0.2)	1 (0.1)	1.00
Antidiabetic	118 (11.3)	81 (9.4)	.17
MoCA, median (IQR)	22.0 (18.0–25.0)	22.0 (18.0–24.0)	.06

Abbreviations: BMI, body mass index; DBP, diastolic blood pressure; DMVs, deep medullary veins; FBG, fasting blood glucose; HbA1C, glycated hemoglobin; HCY, homocysteine; HDL‐C, high‐density lipoprotein cholesterol; IQR, interquartile range; LDL‐C, low‐density lipoprotein cholesterol; MoCA, Montreal Cognitive Assessment; SBP, systolic blood pressure; SD, standard deviation; TIA, transient ischemic attack.

**FIGURE 2:**
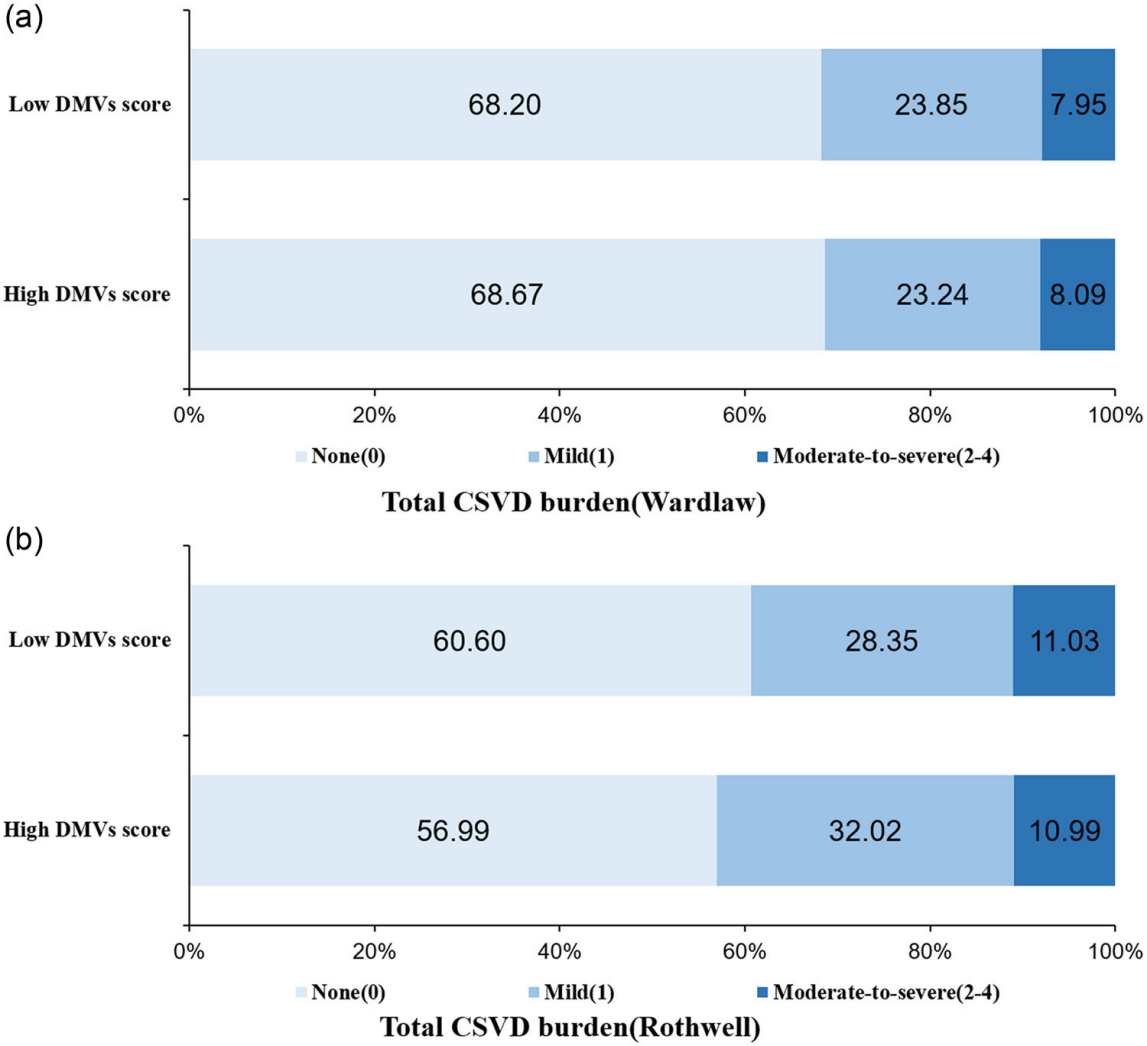
The distribution of CSVD burden according to DMV scores. No significant different distribution of total CSVD burden in two groups divided by the median of DMV scores. CSVD, cerebral small vessel disease; DMVs, deep medullary veins.

Neuroimaging characteristics of CSVD between the two groups divided by DMV scores are presented in Table S4 and Figure 2. According to Wardlaw's scale of total CSVD burden, 31.80% and 31.33% of participants presented with CSVD in the low‐ and high‐DMV‐scores groups, respectively, but there was no statistically significant difference between the two groups (*p* = .83). Similar results were observed according to Rothwell's scale of total CSVD burden (39.94% vs. 43.01%, *p* = .18). The low‐DMV‐scores group had more moderate‐to‐severe CSO‐EPVS compared to the high‐DMV‐scores group (41.67% vs. 37.11%, *p* = .04). No differences in other imaging features, including WMH, lacunae, CMBs, and GCA scale scores, were observed between the two groups.

### 3.2 Associations between vascular risk factors and DMVs

**FIGURE 3:**
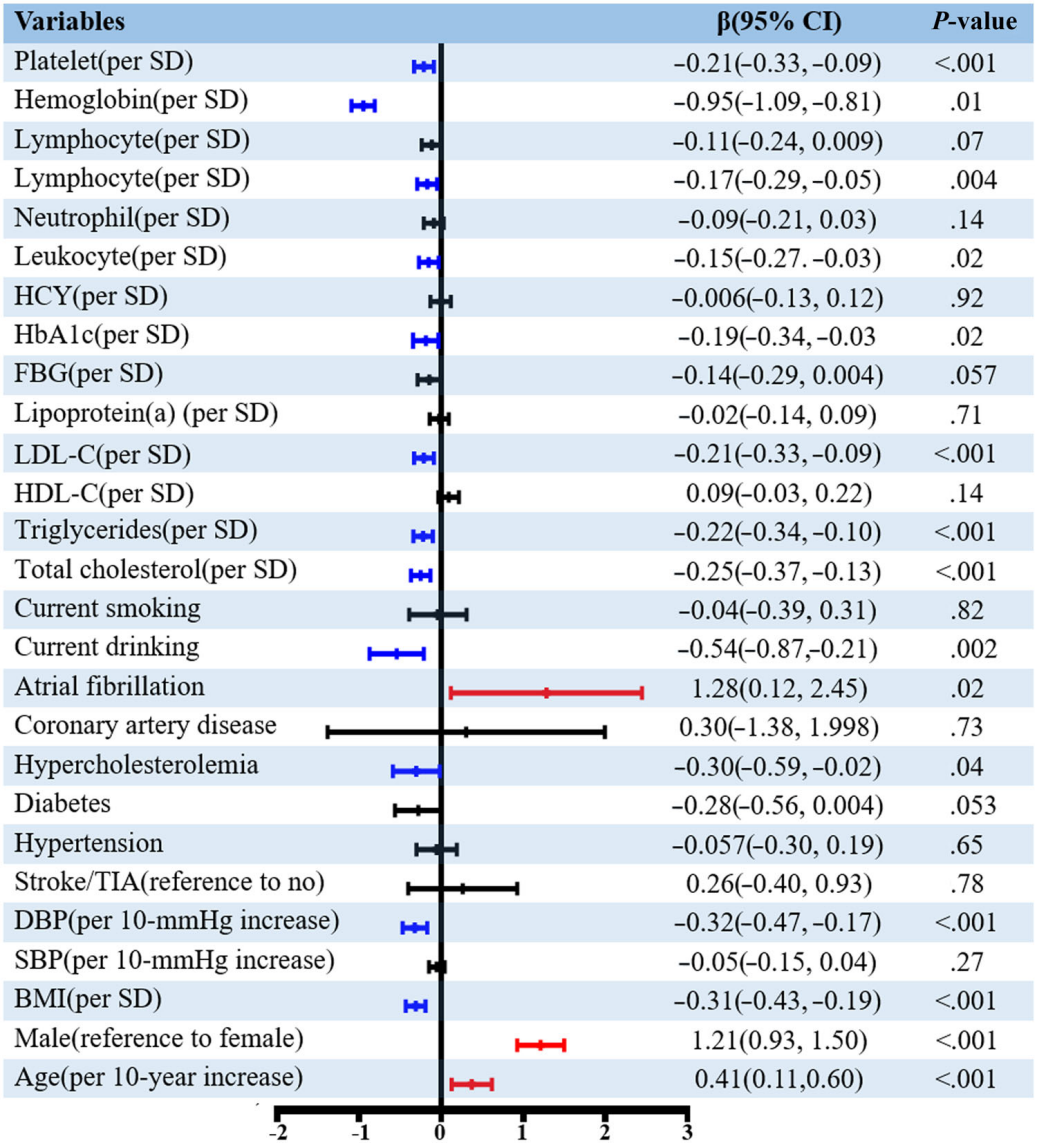
Associations between vascular risk factors and DMV scores. In general linear regression model adjusted for age, sex, BMI, stroke/TIA, hypertension, diabetes mellitus, hypercholesterolemia, coronary artery disease, atrial fibrillation, current drinker, current smoker, and MoCA, the DMVs scores weretreated as the dependent variable, and the potential risk factors were treated as the independent variables. DMVs, deep medullary veins; SD, standard deviation; BMI, body mass index; SBP, systolic blood pressure; DBP, diastolic blood pressure; TIA, transient ischemic attack; HDL, high‐density lipoprotein; LDL, low density lipoprotein; FBG, fasting blood glucose; HbA1C, glycated hemoglobin; HCY, homocysteine; MoCA, Montreal Cognitive Assessment.

Results of univariable and multivariable general linear regression analyses are shown in Table S5. The relative vascular risk factors at a level of *p* < .05 for DMV scores after adjusting for potential confounders in model 3 included age, male sex, BMI, DBP, current drinking, hypercholesterolaemia, AF, TC, TG, low‐density lipoprotein cholesterol (LDL‐C), HbA1c, leukocytes, lymphocyte hemoglobin, and platelets (Figure 3). Older age (*β* = 0.41 per 10 years; 95% CI, 0.11–0.60; *p* < .001) and male sex (*β* = 1.21; 95% CI, 0.93–1.50; *p* < .001) were strongly associated with higher DMV scores. We did not find any evidence of differences according to sex in the association of age with DMV scores (interaction *p*‐value > .05; Table S6).

### 3.3 Associations between DMVs and CSVD

The associations between DMVs and the presence, total burden, and traditional neuroimaging markers of CSVD are presented in Table 2. In crude logistic regression analyses, DMV scores wereassociated with the presence of CSVD (Rothwell's scale) (OR = 1.05; 95% CI, 1.01–1.08; *p* = .009), total CSVD burden (Rothwell's scale) (OR = 1.05; 95% CI, 1.01–1.08; *p* = .02), modified WMH burden (OR = 1.05; 95% CI, 1.01–1.09; *p* = .008), and CSO‐EPVS (OR = 0.96; 95% CI, 0.93–0.99; *p* = .02). However, after adjusting for age and sex in Model 2 and potential confounding factors in Model 3, no significant associations between DMVs and CSVD were identified.

**TABLE 2:** Univariate and multivariate logisticregression for the associations between DMV scores and CSVD

	Model 1^c^		Model 2^c^			Model 3^c^
	cOR/OR (95% CI)	*p*	cOR/OR (95% CI)	*p*	cOR/OR (95% CI)	*p*
Presence of CSVD (Wardlaw)^a^	1.03 (1.00–1.07)	.08	1.02 (0.98–1.06)	.25	>1.03 (0.99–1.07)	.15
Presence of CSVD (Rothwell)^a^	1.05 (1.01–1.08)	.009	1.03 (0.99–1.07)	.10	>1.04 (1.00–1.08)	.06
Total CSVD burden (Wardlaw)^b^	1.03 (0.99–1.07)	.09	1.02 (0.98–1.06)	.31	>1.03 (0.99–1.07)	.22
Total CSVD burden (Rothwell)^b^	1.04 (1.01–1.08)	.02	1.02 (0.99–1.06)	.19	>1.03 (0.99–1.07)	.16
WMH Burden (reference to no)^a^	1.04 (1.00–1.09)	.07	1.01 (0.97–1.06)	.58	>1.02 (0.97–1.07)	.46
Modified WMH burden^b^	1.05 (1.01–1.09)	.008	1.02 (0.98–1.06)	.25	>1.03 (0.99–1.07)	.17
Presence of lacunes^a^	0.96 (0.90–1.04)	.34	0.96 (0.88–1.04)	.28	>0.95 (0.88–1.04)	.27
Presence of CMBs^a^	1.03 (0.97–1.08)	.35	1.03 (0.98–1.09)	.24	>1.03 (0.97–1.09)	.30
CMBs burden^b^	1.03 (0.97–1.08)	.35	1.03 (0.98–1.09)	.24	>1.03 (0.97–1.09)	.30
BG‐EPVS (moderate‐to‐severe)^a^	1.02 (0.97–1.09)	.41	1.01 (0.94–1.07)	.88	>1.00 (0.94–1.07)	.91
CSO‐EPVS (moderate‐to‐severe)^a^	0.96 (0.93–0.99)	.02	0.97 (0.93–1.00)	.07	>0.98 (0.94–1.01)	.18
GCA Scale 2–3^a^	1.02 (0.96–1.08)	.55	0.99 (0.93–1.05)	.69	0.98 (0.92–1.05)	.64

Abbreviations: BG‐EPVS, basal ganglia enlarged perivascular spaces; CMBs, cerebral microbleeds; cOR, common odd ratio; CSO‐EPVS, centrum semiovale enlarged perivascular space; CSVD, cerebral small vessel disease; DMVs, deep medullary veins; GCA scale, global cortical atrophy scale; OR, odd ratio; WMH, white matter hyperintensity.

^a^ In binary logistic regression models, the presence and different imaging markers of CSVD were treated as binary dependent variables, and DMV scores weretreated as independent variable. The ORs were calculated based on per one DMV scores increment.

^b^ In ordinal logistic regression models, total CSVD burden, modified WMH burden, and CMBs burden were treated as ordinal dependent variables, and DMV scores weretreated as independent variable. The cORs were calculated based on per one DMV scores increment.

^c^ Model 1: unadjusted; Model 2: adjusted for age and sex; Model 3: adjusted for age, sex, BMI, stroke/TIA, hypertension, diabetes mellitus, hypercholesterolemia, coronary artery disease, atrial fibrillation, current drinker, current smoker, and MoCA.

## 4 DISCUSSION

In this population‐based study, DMVs were associated with several recognized vascular factors. Age, male sex, and atrial fibrillation were potential risk factors for discontinuous, tortuous, and reduced DMVs. In contrast, BMI, current drinking, DBP, hypercholesterolaemia, plasma lipids (including TC, TG, and LDL‐C), hemoglobin, and blood cells (especially leukocytes, lymphocytes, hemoglobin, and platelets) were potential protective factors. Moreover, DMVs seemed to be weakly associated with total CSVD burden, WMH, and CSO‐EPVS, but no significant differences were observed after adjusting for potential covariates.

Previous studies attempted to identify relative vascular risk factors for DMVs (Ao et al., 2021; Chen et al., 2020), but with the exception of aging, no strong evidence was reported due to small sample sizes or study population limitations. In this study, age was strongly associated with increased DMV scores, implying that aging may result in decreased visibility and discontinuity of DMVs. This was consistent with previous clinical and pathological studies reporting that collagen was deposited in the walls of small periventricular venules, further leading to stenosis and occlusion of veins during the aging process (Ao et al., 2021; Chen et al., 2020; Lin et al., 2017; Moody et al., 1995). DMV scores were higher for men than for women, and men were more likely to have vascular diseases such as cardiovascular disease, stroke, and CSVD. One striking finding of this study was that atrial fibrillation was a vascular risk factor for poorer visible and discrete DMVs. Collagen biomarkers in the circulation, which were fragments of collagen molecules that arose from collagen activity throughout the body, were associated with incident atrial fibrillation (Duprez et al., 2018). Plasma collagen fragments had the opportunity to be deposited in the brain through the blood circulation in patients with atrial fibrillation. Yet, there is no evidence to suggest whether plasma collagen markers were associated with CSVD, and the predictive values of plasma collagen biomarkers for DMVs need to be investigated in the future.

A range of vascular risk factors were identified to have potential protective effects against DMVs. BMI was protective for decreased visibility and discontinuity of DMVs. A phenomenon termed the “obesity paradox” was reported, which suggestedthat underweight individualshad poorer outcomes of cerebrovascular diseases due to progressive catabolic states (Elagizi et al., 2018). A previous study indicated that adiposity reflected by waist circumference was protective for lacunae or CMBs in men (Arnoldussen et al., 2019). However, we did not investigate whether the protective effect of BMI on DMVs differed according to sex. Drinking as a protective factor for DMVs was consistent with the results of previous studies (Gan et al., 2021), in which light‐to‐moderate alcohol consumption reduced the incidence of stroke. Plausible explanations supporting this result are that alcohol beneficially regulates platelet aggregation, fibrinolysis, lipid levels, endothelial function, inflammation, and insulin resistance (Costanzo et al., 2010; Gan et al., 2021). Contrary to previous studies (Ao et al., 2021; Chen et al., 2020), we observed that DBP was negatively associated with reduced visibility of DMVs, and the associations of SBP and hypertension with DMVs were vulnerable. These inconsistent results may be due to the unstable association between blood pressure and pathophysiology in cerebral small veins. Owing to their thin walls and low elasticity, the venules have lower pressure compared to the arterial system (Tucker et al., 2022). It remains unclear whether peripheral arterial blood pressure can reflect structural and functional changes in intracerebral veins. We observed that blood lipid levels, including TC, TG, and LDL‐C, were protective factors for DMVs, whereas HDL cholesterol was a risk factor for DMVs. Although blood lipids are well‐established vascular risk factors for atherosclerosis and stroke, their role in CSVD remains elusive. Previous studies suggested that LDL‐C had no effect on the risk of retinopathy, neuropathy, small vessel stroke, and WMH volume (Georgakis et al., 2020). The differential effects and distinct mechanisms of blood lipids on DMVs, CSVD, and large artery stroke need to be explored in future studies.

Few studies investigated whether systemic inflammation and neuroinflammation wereinvolved in VC. Our results demonstrated that leukocyte and lymphocyte counts were related to DMV scores, implying inflammatory mediation in the progression of VC. A recent study also indicated that inflammatory indicators wereassociated with the number of DMVs (Zhang et al., 2022). An underlying cause is transient adhesion of more leukocytes and lymphocytes to venous vessel walls in the early stage of VC when DMVs are still visible and continuous; however, when brain tissue and vessel wall damagesoccur, this phenomenon disappears (Sienel et al., 2021). Previous studies on blood–brain barrier damage, in which immune cells migrateacross blood vessel walls at postcapillary venules and small veins, also support our findings (Dyrna et al., 2013). While inflammation is involved in atherosclerosis by promoting endothelial dysfunction, our study and a study by Zhang et al. did not observe a correlation between DMVs and HCY, a factor regulating endothelial activation and stability of the cerebrovascular wall (Zhang et al., 2022).

We demonstrated that DMV scores decreased as hemoglobin levels increased, implying better continuity of DMVs. SWI is a blood‐oxygen‐dependent MR sequence that is sensitive to iron in the form of hemosiderin and deoxyhemoglobin (Weber et al., 2021). Through oxygen metabolism in brain tissue, hemoglobin in the blood is converted into deoxyhemoglobin. This results in more visible DMVs on SWI due to increased levels of oxygenated hemoglobin. However, pathological support is required to determine whether the causes of increased DMVs scores were venous occlusion or aggregation of deoxyhemoglobin.

Venous dysfunction in aging and normal white matter diseases drawconsiderable attentions; therefore, we focused on the associations between DMVs and CSVD (Kapadia & Dmytriw, 2021). To our knowledge, this study on the potential role of DMVs in CSVD has the largest sample size and most comprehensive neuroimaging characteristics to date. Our results suggested that DMVs were weakly associated with total CSVD burden, but this relationship was vulnerable after adjusting for confounders. We did not observe associations between DMVs and other common neuroimaging characteristics of CSVD, except for CSO‐EPVS and WMH. This result was in line with a previous study by Ao et al. (2021). However, evidence from small‐sample studies in patients with CSVDindicated that several conventional imaging features of CSVD were correlated with DMVs (Chen et al., 2020; Xu et al., 2020; Zhang et al., 2022, 2019; Zhou et al., 2020). Possible explanations for these discrepancies are as follows. First, differences in the enrolled population and study design may underscore this discordance. In our analysis, all participants from the PRECISE study had a lower prevalence of vascular risk factors and CSVD compared to patients with CSVD, which may have attenuated the relationships between DMVs and diseases. A second potential underlying reason is the different pathogenesis of DMVs and neuroimaging features of CSVD. DMVs on SWI primarily reflect the severity of VC over small veins and venules. In contrast, conventional CSVD markers are predominantly due to small arteriosclerosis (such as fibrinoid necrosis, lipohyalinosis, and microaneurysms) (Pantoni, 2010). Finally, the semiquantitative visual scale of DMVs based on SWI only partially respondsto the pathology of VC at distinct stages of disease. Continuous and visible DMVs on SWI may represent normal veins and venules or compensatory dilated veins due to collagen deposition in the early stage. Further progression of pathology to the decompensated stage as small venous lumens became narrow and occluded, eventually decreasing in number, hypodensity, and signal disruption of DMVs, was observed on SWI. Future studies should more precisely examine the interaction between VC and CSVD in the early or advanced phases of the disease.

This study had several limitations. First, the semiquantitative DMV scale was obtained by observation and comparison rather than by objective measurement. As it was a subjective parameter, there may be bias in the interpretation of images. Second, our findings based on DMV scores that only estimated continuity and visibility of DMVs should be interpreted with caution. The tortuosity of DMVs was more quantitatively described in a small‐sample 7T MRI study (Bouvy et al., 2017). Future studies using 7T MRI should be conducted with larger samples to gain a deeper understanding of the role of DMVs. Third, our analysis was limited to cross‐sectional data of a baseline survey in a community‐dwelling population; therefore, we could not investigate the gradual shrinkage, progressive discontinuity, and eventual disappearance of DMVs. Longitudinal studies should investigate the dynamic changes in DMVs at different stages of CSVD evolution in more detail. Moreover, it was difficult to detect all unmeasured confounding factors that could explain the appearance of DMVs, which is an inherent issue in the present observational study. Finally, this study was conducted exclusively among Chinese patients. These findings warrant further validation in studies involving non‐Asian populations.

## 5 CONCLUSION

We identified several vascular risk and protective factors for decreased visible and discontinuous DMVs in a community‐dwelling population. Our findings offer a new perspective on the potential role of DMVs in normal aging. We did not identify a strong association between DMVs and total burden or traditional imaging markers of CSVD, implying that the pathogenic mechanismsof DMVs and typical imaging markers of CSVD may differ. The structural and functional changes in DMVs during the progression of aging and CSVD warrant further exploration.

## AUTHOR CONTRIBUTIONS

Yilong Wang and Yuesong Pan contributed to the conception and design of the study and provided critical feedback and helped shape the manuscript. All authors contributed to the acquisition and analysis of data. Yu Tian and Shan Li prepared the tables and figures and drafted the manuscript. All authors reviewed the manuscript and approved the submitted version.

## CONFLICT OF INTEREST STATEMENT

The authors declare no conflicts of interest.

### PEER REVIEW

The peer review history for this article is available at https://publons.com/publon/10.1002/brb3.2974.

## Supporting information

Supplementary Table 1. The detailed MRI scan parameters in the present study
Supplementary Table 2. Clinical characteristics in included and excluded participants
Supplementary Table 3. Neuroimaging characteristics of CSVD in included and excluded participants
Supplementary Table 4. Neuroimaging characteristics of CSVD in included participants according the DMV scores
Supplementary Table 5. Linear regression analyses for the associations between potential vascular factors and DMV scores^a^
Supplementary Table 6. Associations of age with DMV scores for men and women

## Data Availability

All data generated or analyzed during this study are included in this published article and available upon reasonable requests.
