# Supplementary material for: Associations of deep medullary veins with vascular risk factors, laboratory indicators, and cerebral small vessel disease: A population‐based study
Source: Brain Behav. 2023 Apr 9;13(5):e2974. doi: 10.1002/brb3.2974 (PMC10176017; doi:10.1002/brb3.2974)
Supplement: Supplementary file 1 — Supplementary Table 1. The detailed MRI scan parameters in the present study Supplementary Table 2. Clinical characteristics in included and excluded participants Supplementary Table 3. Neuroimaging characteristics of CSVD in included and excluded participants Supplementary Table 4. Neuroimaging characteristics of CSVD in included participants according the DMV scores Supplementary Table 5. Linear regression analyses for the associations between potential vascular factors and DMV scoresa Supplementary Table 6. Associations of age with DMV scores for men and women [file BRB3-13-e2974-s001.docx]

**Supplementary Table 1. The detailed MRI scan parameters in the present study**

| **Contrast** | **Orientation** | **Voxel /mm^3^** | **Parameters** | **Scan time** |
| --- | --- | --- | --- | --- |
| T1w MPRAGE | 3D sagittal | 1.00×1.00×1.00 | TE=3.0ms, TR=6.7ms, TI=880ms, shot interval=2000ms, Flip angle=8° | 4:30 |
| T2w | 2D axial | 0.51×0.51×6.50 | TE=105ms, TR=2500ms, SPIR fat suppression | 1:05 |
| SWI | 3D axial | 0.63×0.63×0.80 | first TE=7.2ms, echo spacing = 6.2ms, 5 echoes, Flip angle=17°, TR =37ms | 2:20 |
| FLAIR | 2D axial | 0.53×0.53×6.50 | TE=110ms, TR=7000ms, TI=2300ms, SPIR fat suppression | 2:27 |
| DWI | 2D axial | 1.20×1.20×6.50 | TE=98ms, TR=2500ms, b=1000s/mm^2^, ADC and eADC calculated online | 0:30 |

T1w MPRAGE, T1-weighted magnetization prepared rapid acquisition gradient-echo; T2w, T2-weighted; SWI, susceptibility-weighted imaging; FLAIR, fluid-attenuated inversion recovery; DWI, diffusion weighted imaging; ADC, apparent diffusion coefficient.

**Supplementary Table 2. Clinical characteristics in included and excluded participants**

| **Characteristic** | **Included patients**  **(n=1909)** | **Excluded patients**  **(n=1158)** | **P value** |
| --- | --- | --- | --- |
| **Sociodemographics** |  |  |  |
| Age(years), mean±SD | 61.8±6.5 | 60.3±6.9 | <0.001 |
| Sex(male), n(%) | 1027(53.8) | 613(52.94) | 0.64 |
| BMI(kg/m^2^), median (IQR) | 24.0±3.0 | 23.5±3.2 | <0.001 |
| SBP(mmHg), mean ± SD | 129.0(118.5-140.0) | 127.5(116.5-138.0) | 0.005 |
| DBP(mmHg), mean ± SD | 75.0(69.0-81.0) | 75.0(69.0-81.0) | 0.92 |
| **Vascular risk factors, n(%)** |  |  |  |
| Stroke/TIA | 63(3.3) | 35(3.02) | 0.67 |
| Hypertension | 855(44.8) | 466(40.24) | 0.01 |
| Diabetes Mellitus | 448(23.5) | 215(18.57) | 0.001 |
| Hypercholesterolemia | 427(22.4) | 189(16.32) | <0.001 |
| Coronary artery disease | 9(0.5) | 4(0.35) | 0.81 |
| Atrial fibrillation | 19(1.0) | 7(0.60) | 0.25 |
| Current drinking | 329(17.2) | 245(21.16) | 0.007 |
| Current smoking | 357(18.7) | 272(23.49) | 0.001 |
| **Medication, n (%)** |  |  |  |
| Antihypertensive | 527(27.61) | 296(25.56) | 0.22 |
| Lipid-lowering | 93(4.87) | 27(2.33) | <0.001 |
| Antiplatelet | 59(3.09) | 21(1.81) | 0.03 |
| Anticoagulant | 3(0.16) | 1(0.09) | 0.99 |
| Antidiabetic | 199(10.42) | 75(6.48) | <0.001 |
| **MoCA, median (IQR)** | 22.00(18.00-25.00) | 21.00(17.00-24.00) | <0.001 |

DMVs, deep medullary veins; SD, standard deviation; IQR, interquartile range; BMI, body mass index; SBP, Systolic blood pressure; DBP, Diastolic blood pressure; TIA, transient ischemic attack; MoCA, Montreal Cognitive Assessment.

**Supplementary Table 3. Neuroimaging characteristics of CSVD in included and excluded participants**

| **Characteristics, n(%)** | **Included patients**  **(n=1909)** | **Excluded patients**  **(n=1158)** | **P value** |
| --- | --- | --- | --- |
|  |  |  |  |
| Presence of CSVD(Wardlaw) | 603(31.6) | 331(28.7) | 0.09 |
| Presence of CSVD(Rothwell) | 789(41.3) | 482(41.8) | 0.81 |
| Total CSVD burden(Wardlaw) |  |  | 0.051 |
| None(0) | 1306(68.4) | 823(71.3) |  |
| Mild(1 score) | 450(23.6) | 229(19.8) |  |
| Moderate-to-severe(2-4 score) | 153(8.0) | 102(8.8) |  |
| Total CSVD burden(Rothwell) |  |  | 0.82 |
| None(0 score) | 1120(58.9) | 672(58.5) |  |
| Mild(1 score) | 573(30.1) | 357(31.1) |  |
| Moderate-to-severe(2-4 score) | 208(10.9) | 120(10.4) |  |
| WMH Burden | 322(16.9) | 188(16.3) | 0.68 |
| Modified WMH Burden |  |  | 0.30 |
| 0 | 1253(65.6) | 731(63.3) |  |
| 1 | 569(29.8) | 359(31.1) |  |
| 2 | 87(4.6) | 64(5.6) |  |
| Presence of lacunae | 100(5.2) | 70(6.0) | 0.34 |
| Presence of CMBs | 220(11.5) | 93(8.0) | 0.002 |
| CMBs Burden |  |  | 0.008 |
| 0 | 1689(88.5) | 1064(92.0) |  |
| 1 | 194(10.2) | 82(7.1) |  |
| 2 | 26(1.4) | 11(1.0) |  |
| BG-EPVS(moderate-to-severe) | 180(9.4) | 120(10.4) | 0.39 |
| CSO-EPVS(moderate-to-severe) | 756(39.6) | 394(34.1) | 0.002 |
| Brain atrophy |  |  | <0.001 |
| GCA Scale 0~1 | 1712(89.7) | 1095(94.6) |  |
| GCA Scale 2~3 | 197(10.3) | 63(5.4) |  |

CSVD, cerebral small vessel disease; WMH, white matter hyperintensity; CMBs, cerebral microbleeds; EPVS, enlarged perivascular spaces; BG-EPVS, EPVS in basal ganglia; CSO-EPVS, EPVS in centrum semiovale; GCA scale, global cortical atrophy scale.

**Supplementary Table 4. Neuroimaging characteristics of CSVD in included participants according the DMVs score**

| **Characteristics, n(%)** | **Low DMV score group**  **(n=1044)** | **High DMV score group**  **(n=865)** | **P value** |
| --- | --- | --- | --- |
|  |  |  |  |
| Presence of CSVD(Wardlaw) | 332(31.8) | 271(31.3) | 0.83 |
| Presence of CSVD(Rothwell) | 417(39.9) | 372(43.0) | 0.18 |
| Total CSVD burden(Wardlaw) |  |  | 0.95 |
| None(0) | 712(68.2) | 594(68.7) |  |
| Mild(1 score) | 249(23.9) | 201(23.2) |  |
| Moderate-to-severe(2-4 score) | 83(8.0) | 70(8.1) |  |
| Total CSVD burden(Rothwell) |  |  | 0.23 |
| None(0 score) | 627(60.1) | 493(57.0) |  |
| Mild(1 score) | 296(28.4) | 277(32.0) |  |
| Moderate-to-severe(2-4 score) | 115(11.1) | 93(10.8) |  |
| WMH Burden | 182(17.4) | 140(16.2) | 0.47 |
| Modified WMH Burden |  |  | 0.52 |
| 0 | 697(66.8) | 556(64.3) |  |
| 1 | 301(28.8) | 268(31.0) |  |
| 2 | 46(4.4) | 41(4.7) |  |
| Presence of lacunae | 59(5.7) | 41(4.7) | 0.37 |
| Presence of CMBs | 116(11.1) | 104(12.0) | 0.53 |
| CMBs Burden |  |  | 0.71 |
| 0 | 928(88.9) | 761(88.0) |  |
| 1 | 101(9.7) | 93(10.8) |  |
| 2 | 15(1.4) | 11(1.3) |  |
| BG-EPVS(moderate-to-severe) | 92(8.8) | 88(10.2) | 0.31 |
| CSO-EPVS(moderate-to-severe) | 435(41.7) | 321(37.1) | 0.04 |
| Brain atrophy |  |  | 0.73 |
| GCA Scale 0~1 | 934(89.5) | 778(89.9) |  |
| GCA Scale 2~3 | 110(10.5) | 87(10.1) |  |

DMVs, deep medullary veins; CSVD, cerebral small vessel disease; WMH, white matter hyperintensity; CMBs, cerebral microbleeds; EPVS, enlarged perivascular spaces; BG-EPVS, EPVS in basal ganglia; CSO-EPVS, EPVS in centrum semiovale; GCA scale, global cortical atrophy scale.

**Supplementary Table 5. Linear regression analyses for the associations between potential vascular factors and DMVs score^a^**

|  | **Model 1**^b^ | | **Model 2**^b^ | | **Model 3**^b^ | |
| --- | --- | --- | --- | --- | --- | --- |
|  | **β(95%CI)** | **P value** | **β(95%CI)** | **P value** | **β(95%CI)** | **P value** |
| Age(per 10-year increase) | 0.39(0.20, 0.57) | <0.001 | 0.43(0.26, 0.61) | <0.001 | 0.41(0.11,0.60) | <0.001 |
| Male(reference to female) | 1.31(1.08, 1.55) | <0.001 | 1.34(1.11, 1.58) | <0.001 | 1.21(0.93, 1.50) | <0.001 |
| BMI(per SD) | -0.35(-0.47, -0.23) | <0.001 | -0.33(-0.44, -0.21) | <0.001 | -0.31(-0.43, -0.19) | <0.001 |
| SBP(per 10-mmHg increase) | -0.04(-0.12, 0.03) | 0.25 | -0.10(-0.18, -0.029) | 0.006 | -0.05(-0.15, 0.04) | 0.27 |
| DBP(per 10-mmHg increase) | -0.52(-0.65, -0.38) | <0.001 | -0.37(-0.50,-0.24) | <0.001 | -0.32(-0.47, -0.17) | <0.001 |
| Stroke/TIA | 0.36(-0.32, 1.03) | 0.34 | 0.40(-0.25, 1.05) | 0.22 | 0.26(-0.40, 0.93) | 0.78 |
| Hypertension | -0.10(-0.35, 0.14) | 0.40 | -0.25(-0.50, -0.019) | 0.03 | -0.057(-0.30, 0.19) | 0.65 |
| Diabetes | -0.34(-0.62, -0.057) | 0.02 | -0.42(-0.69, -0.14) | 0.003 | -0.28(-0.56, 0.004) | 0.053 |
| Hypercholesterolemia | -0.19(-0.48, 0.098) | 0.20 | -0.38(-0.66, -0.10) | 0.007 | -0.30(-0.59, -0.02) | 0.04 |
| Coronary artery disease | 0.31(-1.43, 2.07) | 0.72 | 0.37(-1.33, 2.06) | 0.67 | 0.30(-1.38, 1.998) | 0.73 |
| Atrial fibrillation | 0.93(-0.28, 2.14) | 0.13 | 1.08(-0.091, 2.25) | 0.07 | 1.28(0.12, 2.45) | 0.02 |
| Current drinking | -1.07(-1.38, -0.75) | <0.001 | -0.49(-0.82, -0.17) | 0.003 | -0.54(-0.87,-0.21) | 0.002 |
| Current Smoking | -0.99(-1.30, -0.69) | <0.001 | -0.064(-0.41, 0.29) | 0.72 | -0.04(-0.39, 0.31) | 0.82 |
| Total cholesterol(per SD) | -0.17(-0.29, -0.053) | 0.005 | -0.28(-0.40, -0.17) | <0.001 | -0.25(-0.37, -0.13) | <0.001 |
| Triglycerides(per SD) | -0.31(-0.43, -0.19) | <0.001 | -0.31(-0.43, -0.19) | <0.001 | -0.22(-0.34, -0.10) | <0.001 |
| HDL-C(per SD) | 0.29(0.17, 0.41) | <0.001 | 0.17(0.048, 0.28) | 0.006 | 0.09(-0.03, 0.22) | 0.14 |
| LDL-C(per SD) | -0.18(-0.30, -0.057) | 0.004 | -0.23(-0.35, -0.12) | <0.001 | -0.21(-0.33, -0.09) | <0.001 |
| Lipoprotein(a) (per SD) | 0.02(-0.10, 0.14) | 0.33 | 0.0009(-0.12,0.12) | 0.99 | -0.02(-0.14, 0.09) | 0.71 |
| FBG(per SD) | -0.20(-0.32, -0.075) | 0.001 | -0.22(-0.34, -0.11) | <0.001 | -0.14(-0.29, 0.004) | 0.057 |
| HbA1c(per SD) | -0.19(-0.31, -0.07) | 0.002 | -0.24(-0.36, -0.13) | <0.001 | -0.19(-0.34, -0.03 | 0.02 |
| HCY(per SD) | -0.16(-0.28, -0.04) | 0.009 | -0.0069(-0.13, 0.12) | 0.91 | -0.006(-0.13, 0.12) | 0.92 |
| Leukocyte(per SD) | -0.34(-0.46, -0.22) | <0.001 | -0.23(-0.35, -0.11) | <0.001 | -0.15(-0.27. -0.03) | 0.02 |
| Neutrophil(per SD) | -0.25(-0.37, -0.13) | <0.001 | -0.15(-0.27, -0.04) | 0.01 | -0.09(-0.21, 0.03) | 0.14 |
| Lymphocyte(per SD) | -0.27(-0.39, -0.15) | <0.001 | -0.24(-0.36, -0.12) | <0.001 | -0.17(-0.29, -0.05) | 0.004 |
| Monocyte(per SD) | -0.35(-0.46, -0.23) | <0.001 | -0.18(-0.30, -0.06) | 0.003 | -0.11(-0.24, 0.009) | 0.07 |
| Hemoglobin(per SD) | -1.08(-1.19, -0.97 | <0.001 | -1.02(-1.15, -0.88) | <0.001 | -0.95(-1.09, -0.81) | <0.01 |
| Platelet(per SD) | -0.17(-0.29, -0.05) | 0.005 | -0.23(-0.35, -0.12) | <0.001 | -0.21(-0.33, -0.09) | <0.001 |

DMVs, deep medullary veins; SD, standard deviation; CI, confidence interval; BMI, body mass index; SBP, Systolic blood pressure; DBP, Diastolic blood pressure; TIA, transient ischemic attack; HDL, high-density lipoprotein; LDL, low density lipoprotein; FBG, fasting blood glucose; HbA1C, glycated hemoglobin; HCY, homocysteine; MoCA, Montreal Cognitive Assessment.

^a^In the general linear regression models, the DMVs score was treated as the dependent variable, and the potential risk factors were treated as the independent variables.

^b^Model 1: unadjusted; Model 2: adjusted for age and sex; Model 3: adjusted for age, sex, BMI, stroke/TIA, hypertension, diabetes mellitus, hypercholesterolemia, coronary artery disease, atrial fibrillation, current drinking, current smoking and MoCA.

**Supplementary Table 6. Associations of age with DMVs for men and women**

|  | **Model 1** | | **Model 2** | |
| --- | --- | --- | --- | --- |
|  | **β(95%CI)** | **P value** | **β(95%CI)** | **P value** |
| Age (per 10-year increase)^a^ | 0.43(0.26, 0.61) | <0.001 | 0.41(0.22, 0.60) | <0.001 |
| Male(reference to female)^b^ | 1.34(1.11, 1.58) | <0.001 | 1.21(0.93, 1.50) | <0.001 |
| Age × Sex interaction term^c^ | -0.40(-0.76, -0.04) | 0.03 | -0.29(-0.65, 0.07) | 0.12 |
| Male sex | 0.65(0.38, 0.92) | <0.001 | 0.50(0.21, 0.79) | <0.001 |
| Female sex | 0.25(0.01, 0.49) | 0.04 | 0.32(0.06, 0.58) | <0.001 |

DMVs, deep medullary veins; BMI, body mass index; TIA, transient ischemic attack; MoCA, Montreal Cognitive Assessment.

^a^Model 1: adjusted for age; Model 2: adjusted for age, BMI, stroke/TIA, hypertension, diabetes mellitus, hypercholesterolemia, coronary artery disease, atrial fibrillation, current drinking, current smoking and MoCA.

^b^Model 1: adjusted for sex; Model 2: adjusted for adjust for sex, BMI, stroke/TIA, hypertension, diabetes mellitus, hypercholesterolemia, coronary artery disease, atrial fibrillation, current drinking, current smoking and MoCA.

^c^Model 1: adjusted for age, sex, age × sex; Model 2: adjusted for age, sex, age × sex, BMI, stroke/TIA, hypertension, diabetes mellitus, hypercholesterolemia, coronary artery disease, atrial fibrillation, current drinking, current smoking and MoCA.
